# Early-Life Exposure of Pigs to Topsoil Alters miRNA and mRNA Expression in Peripheral Blood Mononuclear Cells

**DOI:** 10.3389/fgene.2022.886875

**Published:** 2022-08-23

**Authors:** M. M. De Souza, D. A. Koltes, H. Beiki, M. A. Sales, T. Tsai, C. V. Maxwell, J. Zhao, J. E. Koltes

**Affiliations:** ^1^ Department of Animal Science, Iowa State University, Ames, IA, United States; ^2^ Department of Animal Science, University of Arkansas-Division of Agriculture, Fayetteville, AR, United States

**Keywords:** immune system, peripheral blood mononuclear cells, livestock, microbiota, RNA sequencing, hygiene hypothesis

## Abstract

Exposure to less-hygienic conditions during early childhood has been associated with stimulation and development of the immune system. A recent study indicated that exposure of piglets to soil-borne microbes during lactation was related with modulation of gut microbiota and immune function. To identify the potential molecular mechanisms and pathways impacted by early-life topsoil exposure, we analyzed the messenger RNA (mRNA) and micro-RNA (miRNA) expression in peripheral blood mononuclear cells (PBMCs) from these piglets. Total RNA was extracted from the PBMCs of piglets exposed to topsoil only from d 4–d 21 of life (mRNA *n* = 6; miRNA *n* = 5) or unexposed control pigs (mRNA *n* = 6; miRNA *n* = 8) at 11, 20, and 56 days of age. Small RNA and mRNA were sequenced with 50-bp single-end reads using Illumina chemistry. Sequence data were quality checked with FASTQC software and aligned to the Sscrofa 11.1 genome with the STAR aligner for mRNA and mirDeep2 for miRNA. Differential expression (DE) analysis was performed using PROC Glimmix of SAS to evaluate changes in expression due to topsoil exposure over time with genes declared DE at a false discovery rate (FDR) of q < 0.10. A total of 138 mRNA and 21 miRNAs were identified as DE for the treatment by age interaction. Ontology enrichment analysis of DE mRNA revealed Gene ontology (GO) terms directly involved in the connection between T-cell and antigen-presenting cells that are associated with T-cell activation. Key regulatory genes identified include *PTPRJ, ITGB3, TRBV30, CD3D*, mir-143, mir-29, and mir-148a. While these results require validation, this study provides data supporting the hypothesis that less-hygienic environments during early life may contribute to the development of the immune system.

## Introduction

Disease resistance of individuals exposed to less-hygienic conditions during early life has been called the hygiene hypothesis, first postulated by Strachan (1989). It proposes that individuals exposed to less-hygienic conditions early in life may have altered immune system development which lowers the incidence of illness later in life (Strachan, 1989). While it is well understood that particular conditions, such as poor water sanitation, lead to the development and propagation of diseases, low environmental exposure has been associated with increased resistance to human chronic illnesses, such as asthma and allergies (Illi et al., 2001; MacNeill et al., 2013; Huang et al., 2021). It has been theorized that this low dose of exposure results in a mild stimulation of the immune system, particularly the adaptive immune system (Su et al., 2013).

A growing number of publications indicate that microbe exposure may be a significant contributor to the development of the immune system (reviewed by Zheng et al., 2020). Ennamorati et al. (2020) identified the gut microbiome as a major presenter of novel antigens to the immune system that influences the generation of regulatory T cells by the thymus in neonatal mice. The authors described how this process may facilitate homeostasis between microbiota and the host immune system in the host to prevent intestinal inflammation later in life. Studies in the chicken also indicate that intestinal microbiota may regulate T-cell production in the thymus in early life (Cheng et al., 2021). Moreover, postnatal microbial colonization of the body may act as a major stimulus of immune tissue development and modulation of miRNA expression (Masotti, 2012), which contributes to “training” of the immune system (Gury-BenAri et al., 2016). This early training of the immune system and development of microbial memory in early life may allow individuals to overcome an illness more quickly, thus, allowing them to spare energy to partition toward growth. In addition, development of a more robust immune system should reduce antibiotic use and subsequently antimicrobial resistance.

As livestock producers have strived to increase sustainability and growth efficiency of pigs, the environment and hygienic conditions have significantly changed over time. During early commercialized swine production, swine were reared outdoors and exposed to soil, weather, and predators. The move to modern, indoor housing has allowed for decreased exposure to parasites, fecal material, predators, and changes in weather conditions, which greatly improved the overall welfare of swine. It has been observed that early-life exposure of pigs to less-hygienic conditions (i.e., topsoil exposure) has increased growth (Dourmad et al., 2009; Lebret et al., 2011; Wenner et al., 2013; Vo et al., 2017) while decreasing feed intake (Lebret et al., 2011; Wenner et al., 2013). One explanation for these findings is that host exposure to microbes from the environment may influence host physiology.

Initial studies of neonatal pigs exposed to topsoil indicate microbial communities (Vo et al., 2017; Wen et al., 2021) and the immune cell population (Tsai et al., 2016; Wen et al., 2021) are altered later in life in response to early-life topsoil exposure. Both Vo et al. (2017) and Wen et al. (2021) identified higher levels of *Prevotella* at days 13 and 12, respectively, in the gut of piglets exposed to topsoil. In mice, perturbation of *Prevotella* in the gut microbiome resulted in exacerbated intestinal inflammation (Iljazovic et al., 2021). Moreover, Vo et al. (2017), who used the same animals described in this study, identified a higher abundance of *Prevotella* at weaning (d 21) and at day 35 (mid-nursery). Vo et al. (2017) also identified higher levels of *Bacteroidetes* and Ruminococcaceae at day 13 and *Clostridium* and *Clostridium cluster XI*, *Coprococcus*, *Campylobacter*, *Streptococcus*, *Mitsuokella*, *Ruminococcus*, *Dialister*, and *Sarcina* during postweaning at the end of the nursery phase (d 56) in response to topsoil exposure. Interestingly, *Clostridium* species have been associated with colonic regulatory T-cell accumulation and improvement of the local and systemic immune responses (Atarashi et al., 2011). Although these studies demonstrate that the microbiome is altered in pigs exposed to topsoil, it is still unclear which specific microbes are directly impacting the animal’s physiology. Little is known about the molecular mechanisms responsible for impacting animal growth or the immune system in this model. Understanding how the immune system is programmed in response to microbes and the environment is an important step in the development of technologies (e.g., probiotics) that may enhance efficiency in livestock production, and provide basic science knowledge about host–microbiome interactions that are beneficial to improve human health.

The objective of this study was to identify changes in peripheral blood mononuclear cell (PBMC) gene expression and associated pathways in response to early-life topsoil exposure in piglets from a subset of piglets exposed to topsoil from previous studies (Tsai et al., 2016; Vo et al., 2017). Specifically, we sought to determine if there was evidence of early, enhanced, or altered immune system development based on mRNA and miRNA expression profiles using RNA-sequencing (RNA-seq) technology. Transcriptome profiling is a powerful tool to evaluate global changes in gene expression within a tissue. As miRNAs are well-established regulators of gene expression and may facilitate communication between the host and microbiome, we evaluated the relationship between miRNAs and known or predicted mRNA targets (Masotti, 2012; Yuan et al., 2018; Gebert and MacRae, 2019).

## Materials and Methods

### Animal Population and Topsoil Exposure Protocol

A detailed description of the animal trial can be found in Vo et al. (2017). Briefly, animal experiments were conducted at the University of Arkansas-Division of Agriculture Swine Research Unit in Fayetteville, Arkansas under protocols approved by the University of Arkansas Institute of Animal Care and Use Committee #13060. A total of 14 synthetic large white pigs were used for the experiment. Control piglets (control group; *n* = 6 for mRNA and *n* = 8 for miRNA analysis) were reared conventionally in farrowing crates, whereas treatment piglets (topsoil group; *n* = 6 for mRNA and *n* = 5 for miRNA analysis) were exposed daily to fresh topsoil from day 4 postpartum until the end of lactation (d 20). All piglets were weaned and transferred to a nursery facility without soil at d 20. Piglets were weighed and blood samples were collected at 11, 20, and 56 days of age. Additional information about the individual pigs (e.g., sex, weight) is provided in Supplementary Tables S1, S2 for mRNA and miRNA experiments, respectively.

### Blood Collection and Isolation of Peripheral Blood Mononuclear Cells

Whole blood samples (10 ml) were collected through venipuncture via jugular vena cava into EDTA tubes (BD Vacutainer^®^ K2EDTA-coated blood collection tube, Franklin Lakes, NJ) from piglets with median body weight (BW) in each litter at days 11, 20 (weaning), and 56 (the end of nursery) of age. PBMCs were isolated using Histopaque®-1,077 (MilliporeSigma, Burlington, MA) gradient centrifugation according to the manufacturer’s protocol and stored at -80°C until total RNA extraction.

### RNA Isolation and RNA-Sequencing Analysis

Total RNA was extracted with TRIzol^®^ using the Direct-Zol RNA Isolation Kit (Zymo Research Corp.), according to the manufacturer’s protocol. RNA quality was determined using an Experion RNA analysis kit (Biorad, Hurcules, CA) to determine RNA degradation using the RNA quality index (RQI) and RNA quantity was measured by fluorometer using a Qubit (ThermoFisher, Waltham, MA). Total RNA samples that passed quantity (100 ng/uL) and quality criteria (RNA quality indicator, RQI = 5.1–9.8) were utilized for sequencing. The RNA-Seq analysis was conducted using Illumina HiSeq4000 chemistry at the Beijing Genomics Institute (BGI) Americas (BGI, Shenzhen, Guangdong, China). Small RNA and mRNA libraries were constructed from the total RNA samples for 50-bp single-end sequencing.

### Bioinformatics Analyses

Sequence read quality was evaluated with FastQC version0.11.3 (Andrews and Babraham Bioinformatics, 2010) and adaptors and low-quality reads were trimmed using Trim Galore version 0.4.5 (Krueger, 2015). Sequence reads from mRNA libraries were aligned to the Sscrofa 11.1 reference genome using the STAR alignment software version 2.5.4a (Dobin et al., 2013). The number of read counts for each transcript was quantified with FeatureCounts version 2.0.3 (Liao et al., 2014). To determine miRNA expression levels, sequence reads were aligned to the Sscrofa11.1 reference genome using miRDeep2 (Friedländer et al., 2008).

### Differential Expression Analysis

Prior to analysis, data were filtered to remove unexpressed (i.e., genes with 0 read counts) and lowly expressed genes. Lowly expressed genes were defined as those not present in 24 of 36 mRNA or 17 of 39 miRNA samples, and with an average expression >2 reads/sample. To account for differences in library size, all samples were normalized using the 75% Quantile method (Bullard et al., 2010). Differential expression analysis was performed with PROC Glimmix of SAS (SAS Institute Inc., 2013) using a negative binomial distribution to fit the model:

$$y_{ijklmno}=treatment_{i}+lane_{j}+RQI_{kl}parity_{m}+age_{k}+litter\;size_{l}x\;age_{k}+litter\;size_{l}+e_{ijklmno},$$

where y = normalized expression value for each sample k; treatment i = topsoil or control; lane j = sequencing lane 1, 2 or 3; RQI = RNA quality score for each sample k from each individual l; parity m = parity of the piglet’s sow (1–4); age = age of piglet at sample k = d 11, d 20, or d 56; sex n = male or female, litter size = number of piglets within piglet’s litter (range: 5–17), and e = random residual error. In addition, pig was included as a random effect to account for the repeated measure across age. The same model was fit for miRNA, except that only one lane was used for sequencing, so no lane effect was needed. For both the mRNA and miRNA analyses, we focused on identifying genes that changed in expression overtime in response to early-life topsoil exposure (i.e., treatment *vs*. age interaction). To account for multiple testing, q-values were calculated in R using the q-value package (Storey et al., 2019) to determine the false discovery rate (FDR) for gene lists. Significant differentially expressed (DE) genes were declared at q < 0.10.

### Co-Expression Network Analysis

To identify pathways of genes that may be impacted by the early-life topsoil exposure treatment, co-expression network analysis was conducted. Co-expression networks were built separately for the control and topsoil mRNA and miRNA groups, respectively, using the Weighted Gene Co-expression Network Analysis (WGCNA) methods and associated R package (Langfelder and Horvath, 2008). The RNA-seq count data were normalized using the Trimmed Mean of M-values method (TMM) in the edgeR package (Robinson et al., 2009). Four network analyses were performed to compare the network modules (putative pathways) between control and topsoil treatments for both the mRNA and miRNA expression data. Spearman’s correlation coefficients were used to obtain the pairwise similarity between gene expression profiles in a signed co-expression network. The soft threshold chosen was β = 14 for all four analyses performed. The minimum module size chosen was 25 and 5 transcripts per module for mRNA and miRNA networks, respectively.

### miRNA:mRNA Module Interactions

To determine the potential miRNA regulation of mRNA pathways, we identified miRNA modules statistically associated with mRNA modules. Spearman correlations were calculated between eigen values for mRNA and miRNA modules and correlation p-values were adjusted to control the FDR using the R package psych version 1.8.10 (Revelle, 2018). To discover individual mRNA or miRNA that may be regulating pathways, mRNAs and miRNA showing higher connectivity within a module were identified as possible hub genes based on module membership (MM) values. The MM values >0.9 were selected as candidate hub (regulator) genes. We used TargetScan to retrieve the known target genes for each miRNA contained within the modules. Only the correlated target genes for the corresponding miRNA within in the correlated miRNA:mRNA module were kept for ontology enrichment analysis.

### Functional Annotation (Ontology Enrichment) Analysis

Biological processes and molecular pathways that differ in response to early-life topsoil treatment were identified using gene ontology enrichment analysis. Functional analysis was carried out using the human orthologues genes since more complete annotation information is available for human genes. Biomart (Durinck et al., 2009) was used to retrieve *Homo sapiens* orthologues for all expressed genes, where orthologues were defined as DNA sequences with >60% nucleotide similarity. Enriched pathways and biological processes were enriched for the DE gene list and miRNA target gene list using the Cytoscape plugins ClueGO v. 2.5.3 and CluePedia v. 1.5.3 (Bindea et al., 2009). The background (reference genes) used in the enrichment analysis were set as all expressed genes in PBMCs from our samples. Biological process gene ontology (GO) terms (GO tree level ≥5) were considered significantly enriched within the gene list at a *p* ≤ 0.05 Benjamini and Hochberg corrected p-value. After identifying the gene-GO matrix, ClueGo clusters similar GO terms into functional groups based on the association strength between the ontology terms, using a kappa score (≥0.5). Group *p*-values were calculated using the number of unique genes found from the uploaded gene list that were statistically associated with the ontology terms included in the group and the total number of unique genes associated with those terms. *p*-value was adjusted using Benjamini and Hochberg (BH) (p-value < 0.05). Enrichment of KEGG pathway terms from small RNA target genes was conducted using the DIANA-miRPath, v3.0 software (Vlachos et al., 2015). Predicted targets were identified using the DIANA-microT-CDS Targets of miRNAs analysis, with default setting (Micro T threshold = 0.08, *p* < 0.05) and enrichment analysis methods (Fisher’s exact test).

## Results

### Evaluation of Sequence Quality

A total of 16.5 million sequence reads were mapped, on average, per sample for the mRNA libraries to the Sscrofa 11.1 reference genome. A total of 13 million sequence reads were mapped, on average, per sample for the miRNA libraries. After applying zero and low expression filters, a total of 14,316 transcripts were available for differential expression analysis in SAS. All sequencing statistics are available in Supplementary Tables S1, S2.

### Differentially Expressed Genes (mRNA), Enriched Biological Process, and Pathway Annotations Identified in Response to Early-Life Topsoil Exposure

**FIGURE 1:**
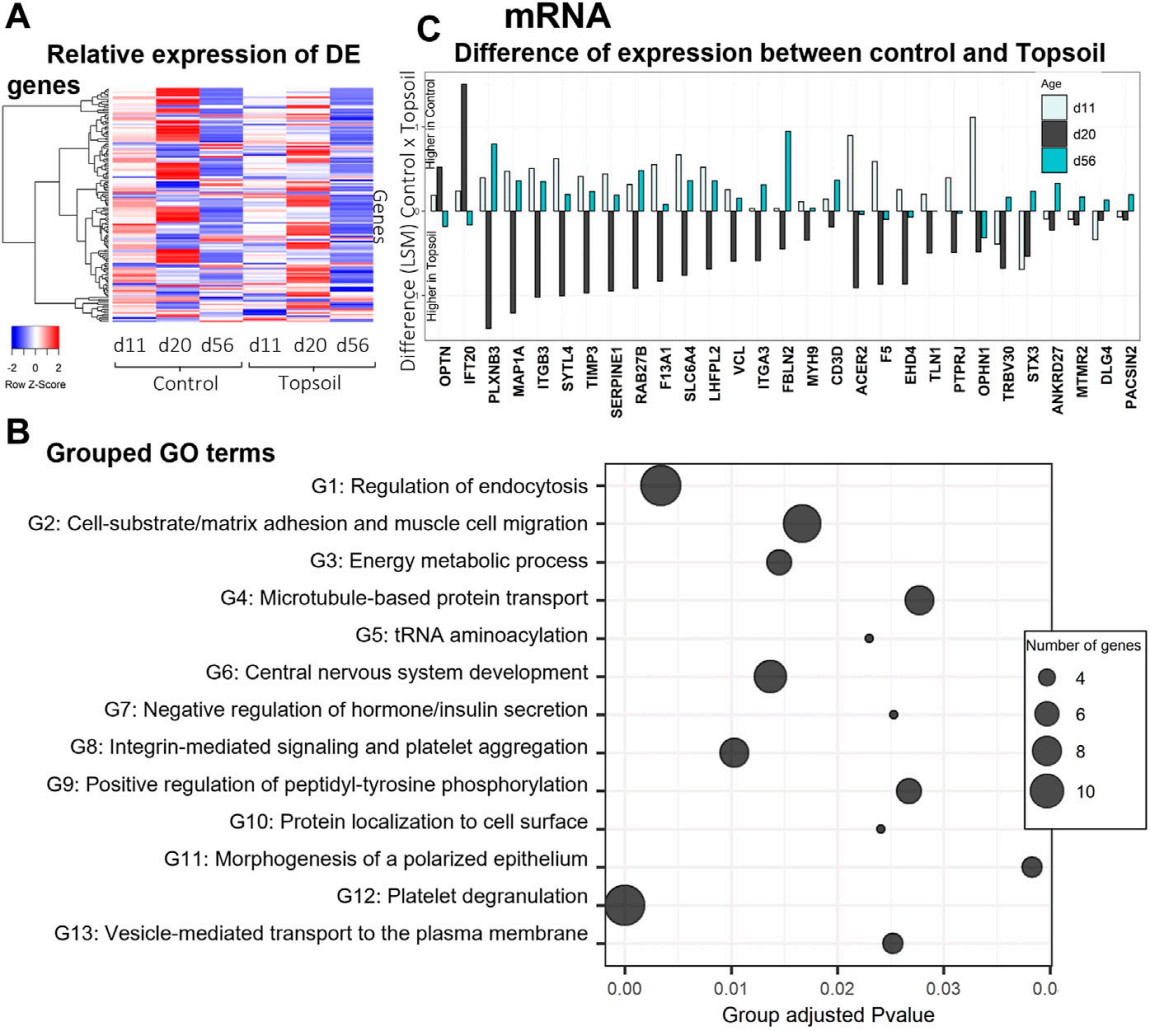
Gene expression and gene ontology (GO) enrichment analysis of the differentially expressed (DE) mRNA in response to topsoil treatment **(A)** A heatmap comparison of the expression of the DE mRNAs across time and treatment (control *vs.* topsoil) in peripheral blood mononuclear cells (PBMCs.). The three timepoints correspond to when pigs were still exposed to topsoil in the nursery (d 11) and at weaning (d 20) as well as after topsoil was removed at the postweaning (d56) timepoint. The heat map shows the clustered relative gene expression (least squares mean–LSM) for control and topsoil treatments by age (day of life/age of piglet: 11, 20, and 56) **(B)** Visualization of the number and significance of grouped gene ontology (GO) terms identified using the DE genes in ClueGo software. The group p-values (provided on the *x*-axis) were calculated using the list of unique DE genes and adjusted using a Benjamini and Hochberg correction (BH). *p*-values were considered significant at BH adjusted p-value < 0.05. The size of the bubbles represents the number of genes clustered in the ontology term group **(C)** A histogram showing the difference in transcript abundance between the control and topsoil treatment at days 11, 20, and 56 for selected genes with ontology terms related to functionality or components of the immune system. Differences in mRNA abundance were estimated using the LSM between control and topsoil treatments at each specific time point (piglet age). Positive differences (above zero) means higher abundance in the control group while negative differences (below zero) mean higher in topsoil group.

A total of 138 genes were identified as DE for the treatment by age interaction (q < 0.10). The full list of DE genes and associated statistics are available in Supplementary Table S3. A heat map displaying the expression levels of DE genes is provided in Figure 1A. Trends in gene expression patterns observed over time when comparing the topsoil treatment to control for all DE genes are shown in Supplementary Figures S1–S6 for all the DE genes. To better understand the function of the DE mRNAs, GO enrichment analysis was performed using the DE genes that had annotations for human orthologues (109 of 138 DE genes). A total of 59 GO terms were identified as statistically enriched for specific biological terms (p-adjusted < 0.05; Figure 1B, Supplementary Table S4). Forty-three of these GO terms were clustered into 13 groups with similar GO term biological processes (BP) (Figure 1B). The most statistically significant ontology term was platelet degranulation (adjusted *p* < 0.01). The three largest clusters of enriched GO terms (Groups 1, 2, and 12; Figure 1B) were involved in processes related to functionality or components of the immune system. Four additional gene ontology groups identified were also known to be involved in immune system, including the terms microtubule-based protein transport (group 4), integrin-mediated signaling pathways (group 8), positive regulation of peptidyl-tyrosine phosphorylation (group 9) and positive regulation of cell-substrate adhesion (group 13). The genes in these seven combined ontology term groups had higher levels of transcript abundance at day 20 in topsoil-treated animals (with the exception of *IFT20* and *OPTN*; Figure 1C). A total of 15 out of 28 genes in these ontology groups exhibited higher levels of transcript abundance in the control piglets at days 11 and 56. Although the DE genes *CD3D* and *TRBV30* were not included in these ontology term groups, they also had higher expression in topsoil-treated piglets at day 20 in the same pattern observed in the seven ontology groups identified. Since *CD3D* and *TRBV30* play a major role during immune response as components of the T-cells receptor (TCR) complex, they are also highlighted in Figure 1C. Other statistically significant ontology terms (adjusted *p* < 0.05) included: negative regulation of insulin secretion, positive chemotaxis, BMP signaling, SMAD signaling, iron ion transport, and homeostasis and glucan metabolic process.

### Differentially Expressed miRNA, Enriched Biological Process, and Pathway Annotations Identified in Response to Early-Life Topsoil Exposure

**FIGURE 2:**
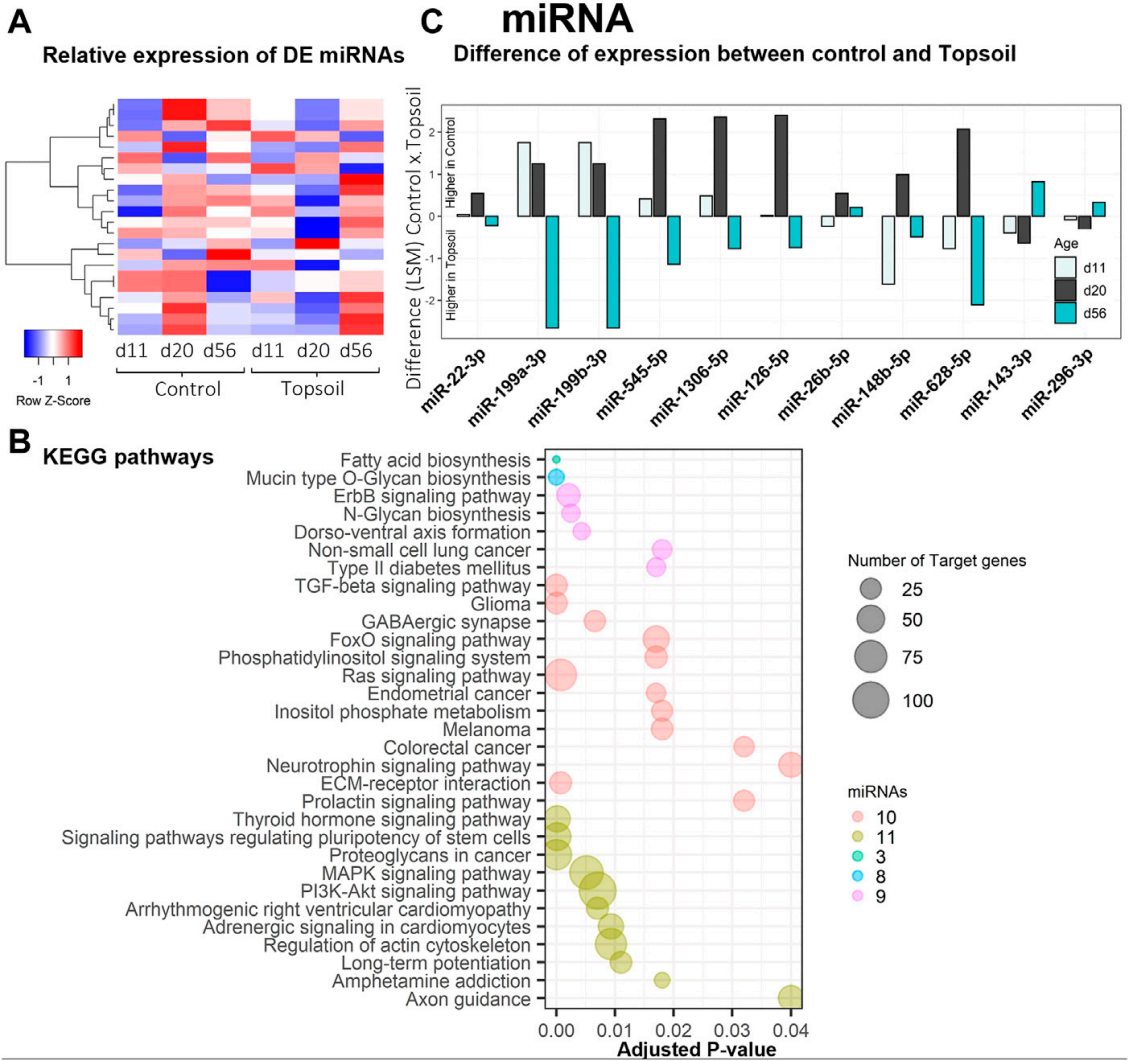
Gene expression and gene ontology (GO) enrichment analysis of the differentially expressed (DE) miRNA response to topsoil treatment **(A)** A heatmap comparison of the expression of the most highly DE miRNAs across time and treatment (control *vs*. topsoil) in peripheral blood mononuclear cells (PBMCs.) The three timepoints correspond to when pigs were still exposed to topsoil in the nursery (d 11) and at weaning (d 20) as well as after topsoil was removed at the postweaning (d56) timepoint. The heat map shows the clustered relative miRNA expression (least squares mean—LSM) for control and topsoil treatments by age (day of life/age of piglet: 11, 20, and 56) **(B)** Visualization of the number and significance of KEGG pathway terms identified by DIANA pathway ontology enrichment analysis of DE miRNA. The size of the bubbles represents the number of target genes clustered in the KEGG ontology term group. Bubbles were colored by the number of miRNAs enriched within an ontology group **(C)** A histogram showing the difference in abundance of miRNA expression between control and topsoil treatment at each time points of miRNAs clustered in the KEGG ontology. Differences in miRNA abundance were estimated using the LSM between control and topsoil treatments at each specific time point (piglet age). Positive differences (above zero) mean higher abundance in the control group while negative differences (below zero) mean higher in topsoil group.

A total of 542 miRNAs were identified by miRDeep2 software. After filtering for annotations, a total of 254 miRNAs were used for the statistical analysis. Twenty-one unique miRNAs were identified as DE for the treatment by age interaction (q < 0.10; miRNA DE results are listed in Supplementary Table S5). A heat map displaying the expression levels of DE miRNA is provided in Figure 2A. Trends in miRNA expression patterns observed over time when comparing treatments are shown in Supplementary Table S7. The five most significantly DE miRNA included miR-1306, miR-660, miR-126, miR-1285, and miR-1468. Notably, six of the miRNAs identified as DE had gene annotations related to functionality or components of the immune system. To better understand the function of the DE miRNAs, enrichment analysis was performed using the KEGG ontology to identify enriched pathways that may be affected by these miRNAs (Figure 2B, Supplementary Table S6). Enrichment analysis identified 32 different pathways associated with these miRNAs. Of these 32 pathways, seven were directly associated with disease phenotypes and seven were associated with cellular signaling (i.e., neurotrophin signaling to Ras pathway signaling). Additional commonly enriched KEGG pathways were neurological signaling (4 pathways) and development (3 pathways). In total, these KEGG pathways included 11 of the DE miRNAs miR-126–5p, miR-1306–5p, miR-143–3p, 148b-5p, miR199a-3p, miR199b-3p, miR-22–3p, miR26b-5p, miR-296–3p, miR545–5p, miR-628–5p. Most of these 11 miRNAs showed higher abundance at day 20 in piglets exposed to topsoil, except for miR-143–3p, miR-296–3p, and miR-22–3p, as shown in Figure 2C.

### Identification of mRNA and miRNA Modules

**FIGURE 3:**
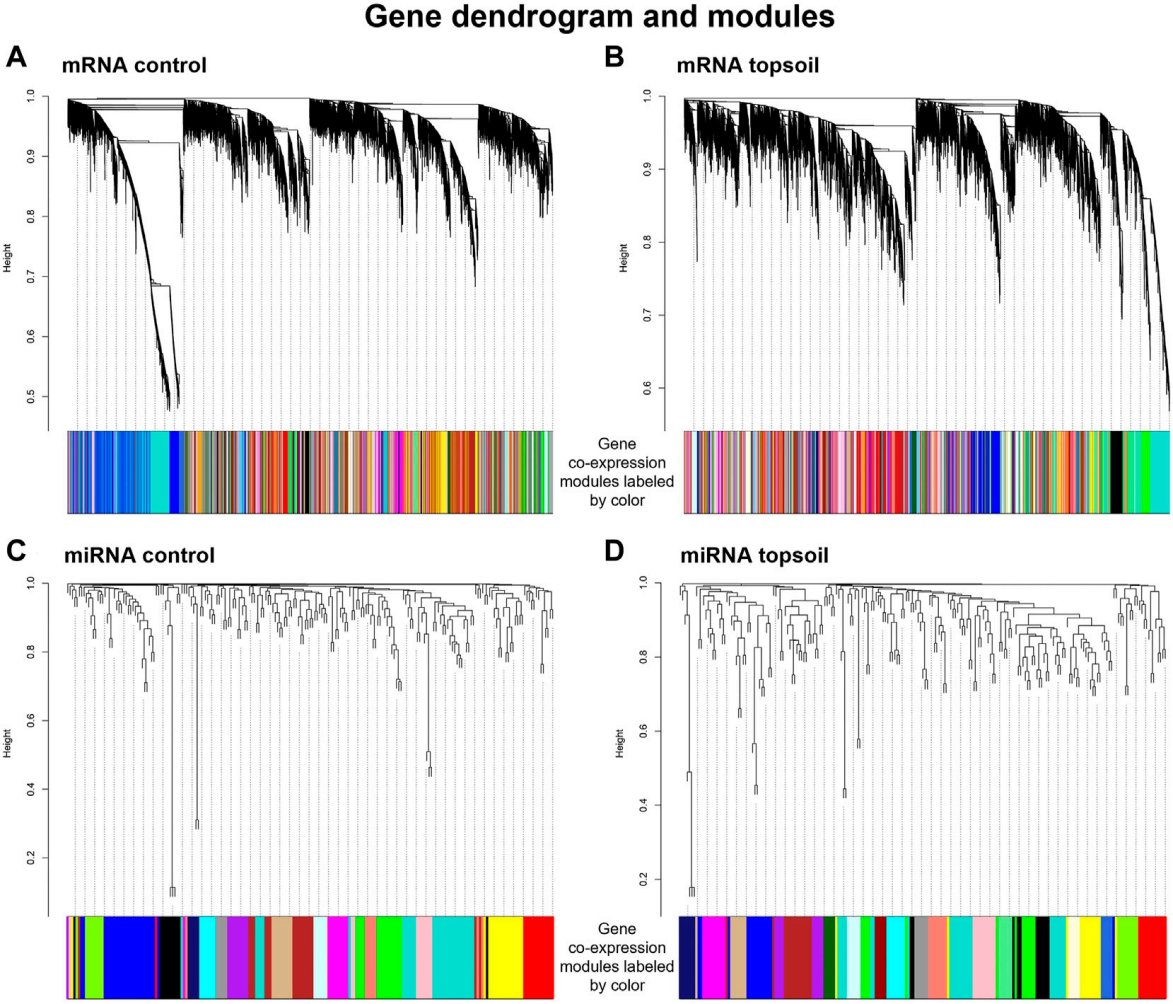
Gene co-expression modules identified for the mRNA control group **(A)**, mRNA topsoil **(B)** miRNA control **(C)** and miRNA topsoil **(D)** groups. Co-expression modules are differentiated by color. Each group of co-expression modules is accompanied with the corresponding gene dendrogram.

To determine coordinated (i.e., correlated) changes in gene expression in response to early-life topsoil exposure, gene co-expression network modules were identified using 14,316 mRNA genes and 185 miRNAs, separately. A total of 99 control mRNA modules and 144 topsoil mRNA modules were identified (Figures 3A,B). A total of 17 control miRNA modules and 22 topsoil miRNA modules were identified (Figures 3C,D). We identified 22 DE genes as hub genes in control modules and 23 in topsoil modules (Supplementary Table S3). For miRNA, four DE miRNA were hub genes in control modules while eight were hub in topsoil modules (Supplementary Table S5).

### Identification of miRNA:mRNA Module Interactions and Functional Analysis of Target Genes

To identify the potential regulation of mRNA by miRNA, miRNA:mRNA modules were identified. A total of 11 miRNA and mRNA module pairs were identified as significantly correlated (q-value < 0.05; Table 1). In the control group, only one pair of negatively correlated modules was identified (miR-blue:mRNA-turquoise). Five negatively and five positively correlated modules were identified in the topsoil group with some modules identified as correlated across multiple modules. For example, the topsoil miR-blue module was correlated to three different mRNA modules. The black, brown and salmon miRNAs modules were each correlated with two different mRNA modules. Only one mRNA module (turquoise) was correlated to multiple miRNA modules in topsoil. Table 2 summarizes the GO BP enrichment results for the four most highly correlated mRNAs modules with miRNA modules in the topsoil group. The complete list of enriched BPs is presented in Supplementary Table S7. Only seven out of 11 miRNA:mRNA pairs enriched for GO terms. The miRblack:black interaction showed the highest number of enriched BP, totalizing 204 BPs clustered in 36 groups, including pathways directly related to immune response, such as the groups lymphocyte homeostasis, natural killer cell-mediated cytotoxicity and regulation of T-cell receptor signaling pathways. The second highest enrichment was for the miRbrown:brown interaction with 36 BPs clustered in 10 groups including immunity-related pathways such as positive regulation of B-cell receptor signaling pathway and response to lipopolysaccharide. All the groups enriched for MiRblack:antiquewhite4 and miRblue:royalblue modules interaction were related to functionality or components of the immune system.

**TABLE 1:** Identification of correlated (q < 0.05) miRNA:mRNA modules. Modules are identified with colors names designated by WGCNA software within treatment group (topsoil or control). The number of miRNA (# miRNAs) and mRNA (# mRNA) within each module, correlation between module pairs (R), and significance of the correlation (q-value) are presented.

Treatment	miRNA Module	# miRNAs	mRNA Module	# mRNA	q-value	R
Control	miR-blue	25	turquoise	1,480	0.0018	−0.921
Topsoil	miR-salmon	8	turquoise	1,234	0.0485	−0.875
	miR-brown	14	brown	739	0.0485	−0.839
	miR-blue	15	royalblue	138	0.0485	−0.835
	miR-blue	15	sienna3	91	0.0485	−0.835
	miR-black	11	black	389	0.0485	−0.832
	miR-red	12	darkseagreen4	60	0.0485	0.825
	miR-salmon	8	thistle1	68	0.0485	0.825
	miR-blue	15	turquoise	1,234	0.0485	0.825
	miR-black	11	antiquewhite4	58	0.0485	0.853
	miR-brown	14	skyblue3	87	0.0485	0.853

**TABLE 2:** Summary of enriched Gene Ontology (GO) biological processes (BP) for four miRNA:mRNA co-expression interaction networks in the topsoil group. miRNA targets contained in the correlated mRNA module were used for the enrichment.

miRNA:mRNA module^a^,^b^	Target	Group GO-Term^c^	Group PValue (BH)^d^	N. BPs	N. Genes
	Genes^$^				
brown:brown	201	Positive regulation of B-cell receptor signaling pathway	0.0330	3	3
		Response to lipopolysaccharide	0.0079	1	12
		Response to progesterone	0.0233	1	3
		Peptide catabolic process	0.0198	1	3
		Negative regulation of DNA-dependent DNA replication	0.0244	1	3
		Regulation of keratinocyte proliferation	0.0233	1	3
		Protein glycosylation	0.0062	3	10
		Monocarboxylic acid catabolic process	0.0180	3	7
		Negative regulation of intracellular protein transport	0.0193	4	7
		Negative regulation of DNA biosynthetic process	0.0055	5	9
		Response to peptide hormone	0.0003	13	23
black:antiquewhite4	24	Negative regulation of cytokine biosynthetic process	2.1E-05	1	3
blue:royalblue	64	Interferon-gamma-mediated signaling pathway	0.0006	1	4
		Defense response to virus	7.2E-06	3	11
		Viral genome replication	0.0004	4	4
black:black	176	Lymphocyte homeostasis	0.0213	1	4
		Natural killer cell-mediated cytotoxicity	0.0241	1	3
		Regulation of T cell receptor signaling pathway	0.0356	1	3
		Virus receptor activity	0.0060	7	10
		Virion assembly	0.0006	9	14
		Protein serine/threonine phosphatase activity	0.0349	1	4
		Blood coagulation, fibrin clot formation	0.0121	1	3
		Regulation of glycoprotein metabolic process	0.0382	1	3
		Vesicle targeting	0.0201	1	5
		Activation of cysteine-type endopeptidase activity involved in apoptotic process	0.0394	1	4
		Neuropeptide signaling pathway	0.0099	1	4
		Regulation of G protein-coupled receptor signaling pathway	0.0346	1	5
		Regulation of cell shape	0.0112	1	7
		Antioxidant activity	0.0357	1	4
		Embryonic digestive tract development	0.0094	1	3
		Muscle filament sliding	0.0127	1	3
		Platelet degranulation	8.1E-06	1	12
		Cyclic nucleotide-dependent protein kinase activity	0.0178	1	3
		Artery development	0.0267	1	4
		Actomyosin structure organization	0.0129	2	10
		Cardiac muscle cell apoptotic process	0.0233	2	3
		Renal water homeostasis	0.0390	2	3
		Protein transport within lipid bilayer	0.0020	4	7
		Myotube differentiation	0.0202	4	6
		Calcium-mediated signaling using intracellular calcium source	0.0197	4	9
		Negative regulation of potassium ion transport	0.0116	4	9
		Platelet aggregation	0.0007	4	13
		Regulation of focal adhesion assembly	0.0201	5	5
		Regulation of muscle contraction	0.0001	6	14
		Rab protein signal transduction	0.0056	6	6
		Regulation of smooth muscle contraction	0.0004	7	10
		Cellular response to amino acid starvation	0.0195	8	10
		Chaperone-mediated protein complex assembly	0.0116	9	9
		Regulation of transmembrane receptor protein serine/threonine kinase signaling	0.0069	12	10
		Histamine secretion	0.0006	22	20
		ADP metabolic process	0.0056	28	15
		Positive regulation of hormone secretion	4.4E-06	42	37

a Individual miRNA, and mRNA, modules (i.e., highly correlated genes based on co-expression analysis) are identified with a unique color name.

b Only the four most highly correlated mRNAs, modules with miRNA, modules in topsoil group are showed in this summary table^**$**^ For each mRNA, module, genes that were the target of the associated miRNA, modules were used for the enrichment analysis in ClueGO.

c Biological process ontology terms directly related to immune function are highlighted in red.

d The group p-value was calculated using the list of unique genes found within the uploaded gene list. *p*-values were adjusted using a Benjamini and Hochberg correction (BH) (p-value < 0.05).

## Discussion

The objective of this study was to identify potential mechanisms controlling how the immune system is programmed in response to microbes and the environment. To understand how early-life exposure to less-hygienic conditions alters immune training, piglets were exposed to topsoil only from d 4-d 21 of life, which resulted in the modulation of gut microbiota and growth performance (Vo et al., 2017). To determine changes in immune signaling, PBMC mRNA and miRNA gene expression were analyzed over the first 56 days of life. The following passages provide potential explanations for the observed changes in PBMC gene expression in response to early-life topsoil exposure along with the potential limitations in the interpretation of this study.

Genes identified as DE indicate changes in the development of the immune system which under the parameters of this study may be in response to interactions with novel antigens or nutrient availability. This analysis identified DE genes associated with T-cell activation. The first two DE genes, *PTPRJ* and *ITGB3,* are known to be involved in the formation of the immune synapse or area of communication between a T cell and an antigen-presenting cell (APC). Both genes were upregulated at d 20 in the topsoil group (Supplementary Figure S1, Figure 1C) which may be in response to increased contact with microbes during the nursery phase from d 4 to d 21. Corroborating these results, Lin et al. (2004) found increased levels of *PTPRJ* in T cells upon activation in mice, indicating that this increase in the topsoil-exposed piglets may have elevated T-cell activation at d 20. Conversely, the lower levels of *PTPRJ* in naïve T cells in mice (Lin et al., 2004) indicate that the lower expression of *PTPRJ* in the control piglets at d 20, when the piglets were in higher hygienic conditions, results from lower antigen contact and probably higher naïve T cells.

Two of the identified DE genes makeup components of the T-cells receptor (TCR) complex, *TRBV30* and *CD3D,* are crucial to antigen recognition and initiation of cellular signaling. The diversity of paired alpha and beta chains of the TCR repertoire confers broad immune coverage against pathogens. Recent studies using next-generation sequencing have characterized the TCR repertoire of individuals. The TCR repertoire is composed of a diverse combination of alpha and beta TCR chains (Attaf et al., 2015), which may provide the pathogen exposure history and immunological memory of a given individual (DeWitt et al., 2018). *TRBV30* is one of the several genes encoding the V region of the variable domain of TCR beta chain which is responsible for recognizing specific peptides presented by APCs (Davis et al., 1995). The *CD3D* gene is associated with TCR chain activation after antigen recognition (Dave et al., 1997; Garcillán et al., 2021). Both *TRBV30* and *CD3D* exhibited similar trends of expression over time in this study. In piglets exposed to topsoil, expression levels increased from d 11 to d 20 and then decreased at d 56 (Figure 1C). In control piglets, expression levels decreased from d 11 to d 20 and increased at d 56. The increased expression at d 20 followed by a decrease at d 56 in the topsoil group as well as the delayed peak in the control piglets may indicate earlier development of the immune system in topsoil-exposed piglets compared with the control. Early-life exposure to topsoil may have provided microbes or other substances that resulted in these differences in gene expression that allow for faster response to future exposure of these microbes. Once ingested by the piglets, microbes and other constituents of the topsoil would interact with Paneth cells and APCs that stimulate the immune system.

Other DE genes with known function in immune response (i.e., *BMP8A, GPD2*, *PHKA1* and *PHKA2*) (Supplementary Figure S1) exhibited a similar pattern of expression, which may support the hypothesis that exposure to topsoil alters the timing or development of the immune system. Of particular interest, *BMP8A* is a transforming growth factor beta (TGF-β) superfamily member contained within a signaling pathway essential to activation and homeostasis of naïve CD4^+^ T cells (Martínez et al., 2015).

The ontology enrichment analyses identified pathways involved in T-cell activation, specifically those involved in the interaction between the T cell and the APC, including the formation of the interactive space between the two also known as the immunological synapse. The DE genes *STX3, PTPRJ*, *ITGB3, ITGA3*, *MYH9*, and *TLN1* were clustered in several GO terms related to processes involved in many aspects of the immune synapse and downstream signaling. These genes function in vesicle-mediated transport to the plasma membrane (GO:0098876), protein transport within plasma membrane (GO:0099632), microtubule-based protein transport (GO:0099118), integrin-mediated signaling pathways (GO:0007229), positive regulation of peptidyl-tyrosine phosphorylation (GO:0050731), and positive regulation of cell-substrate adhesion (GO:0010811). After T-cell receptor stimulation, cytoskeleton reorganization (actin and microtubules) is needed to adapt the cell conformation and formation of the synapse architecture (Valitutti et al., 1995; Martín-Cófreces and Sánchez-Madrid, 2018). Traffic through the membrane is important to regulate the activation of T cells by balancing the cellular localization of a wide type of components as receptors, signaling molecules or cytokines (reviewed by Soares et al., 2013). *STX3* has a putative role in the formation of the immune synapse of cytotoxic T cells as it is involved in protein trafficking involving vesicle fusion and exocytosis. STX3 co-localized with CD3 proteins which are components of TCR-CD3 complex on T-cell surfaces (Pattu et al., 2012). Two additional DE genes, *PTPRJ* and *ITGB3,* are known to be involved in the formation of the immune synapse and clustered in GO terms related to cell-substrate adhesion and regulation of tyrosine phosphorylation. The *ITGB3* gene plays a role in the immune synapse by encoding a subunit of specific integrins. Other DE genes involved in these processes include *ITGA3*, *MYH9,* and *TLN1* (Figure 1C, Supplementary Figure S1), all of which increase at d 20 in piglets exposed to topsoil suggesting that these genes support the formation of the immune synapse (Figure 1C).

An important finding in relation to the development of the immune system was the identification of mir-143 as DE in response to topsoil exposure over time (Figure 2C). MicroRNAs are important regulators of gene expression with important roles in mediating the function of the immune system (Ma et al., 2011; Zhang et al., 2018). Typically, each miRNA suppresses gene expression in tens to thousands of mRNA transcripts to coordinate the use of pathways within the transcriptome (Gebert and MacRae, 2019). Previous studies indicate that overexpression of mir-143 may enhance the conversion of cytotoxic T cells into memory cells by reprogramming cellular metabolism (Zhang et al., 2018). In addition, mir-143 was identified as a candidate regulator of a miRNA-target co-expression network in response to topsoil and a hub miRNA within the brown miRNA module. It was also negatively correlated with a group of mRNAs (brown mRNA module) only in the piglets exposed to topsoil during early life. Target mRNA genes within the miRNA:mRNA correlated modules containing mir-143 were enriched for biological processes and pathways such as response to lipopolysaccharide and positive regulation of B-cell receptor signaling pathway. Together, mir-143, the other hub miRNAs, and their co-expressed target genes in the brown module make up a miRNA-target network acting as a putative driver of the immune response in the animals exposed to topsoil in early life.

Analysis of mRNA-miRNA interactions also identified mir-29a-3p and mir-148–5p as potential regulators of gene expression only in topsoil-exposed pigs. Upon bacterial infection, mir-29a-3p has been identified to be downregulated in innate immune cells, such as natural killer and adaptive immune cells such as helper T cells and cytotoxic T cells (Ma et al., 2011). Targets of mir-29a-3p were enriched for regulation of mTOR signaling which has a role in the innate and adaptive immune system by regulating metabolism. As reviewed by Chi. (2012), mTOR signaling can act in different pathways affecting T-cell activation, differentiation, and immune homeostasis. The third interesting candidate miRNA-target network identified was driven by mir-148a-5p, which was negatively correlated to the royalblue module. The mRNA genes within the royalblue mRNA module were enriched for genes involved in response to type I interferon and interferon-gamma-mediated signaling pathway. Type I interferon is secreted by the innate immune cells after detection of the pathogen, but also can act in the adaptive immune response by stimulating antibody production of B cells or intensifying the T-cells function (Reviewed by Ivashkiv and Donlin, 2014).

## Conclusion

Understanding the role hygienic environments have on host gene expression is important in our understanding of the development of the immune system. The findings of this study indicate that early-life exposure of piglets to topsoil impacts both mRNA and miRNA expression in PBMCs, particularly genes and miRNA involved in the development of T cells and their ability to interact with antigen-presenting cells. Key regulatory genes identified include: *PTPRJ, ITGB3, TRBV30, CD3D*, mir-143, mir-29, and mir-148a. While these results need to be interpreted with caution as they represent only a snapshot in time across a small window of time from a subset of peripheral immune cells, these results support the current hygiene hypothesis in that less-hygienic environments during early life contribute to the development of the immune system. However, additional studies are needed to characterize how topsoil exposure may impact gene expression within the gut and impact gut phenotypes critical to animal health.

## Data Availability

The RNA-Seq data set generated and analyzed during the current study is available in the NCBI’s Gene Expression Omnibus (GEO) under accession numbers GSE205321.

## Abbreviations

APC, antigen-presenting cell; BP, biological process; DE, differentially expressed; FDR, false discovery rate; GO, gene ontology; MM, module membership; PBMC, peripheral blood mononuclear cells; RNA-Seq, RNA sequencing; RQI, RNA quality score; TCR, T-cells receptor; TMM, Trimmed Mean of M-values; WGCNA, the Weighted Gene Co-expression Network Analysis.
