# Supplementary material for: Early-Life Exposure of Pigs to Topsoil Alters miRNA and mRNA Expression in Peripheral Blood Mononuclear Cells
Source: Front Genet. 2022 Aug 23;13:886875. doi: 10.3389/fgene.2022.886875 (PMC9445269; doi:10.3389/fgene.2022.886875)
Supplement: Supplementary file 1 [file Presentation1.zip › Supplementary Figures.docx]

**Early life exposure of pigs to topsoil alters miRNA and mRNA expression in peripheral blood mononuclear cells**

**M. M. de Souza^1^, D. A. Koltes^1^, H. Beiki^1^, M. A. Sales^2^, T. Tsai^2^, C. V. Maxwell^2^, J. Zhao^2^, and J. E. Koltes^1*^**

^1^Department of Animal Science, Iowa State University, Ames, IA, 50011, USA

^2^Department of Animal Science, University of Arkansas-Division of Agriculture, Fayetteville, AR, 72701, USA

**Supplementary Figures** below display the change in gene expression in piglet periferal blood mononuclear cells (PBMCs) observed over time for the differentially expressed (DE) mRNA and miRNA (q < 0.10) in the comparison of topsoil exposure during early life (d4 to d20) compared to control (no exposure.)


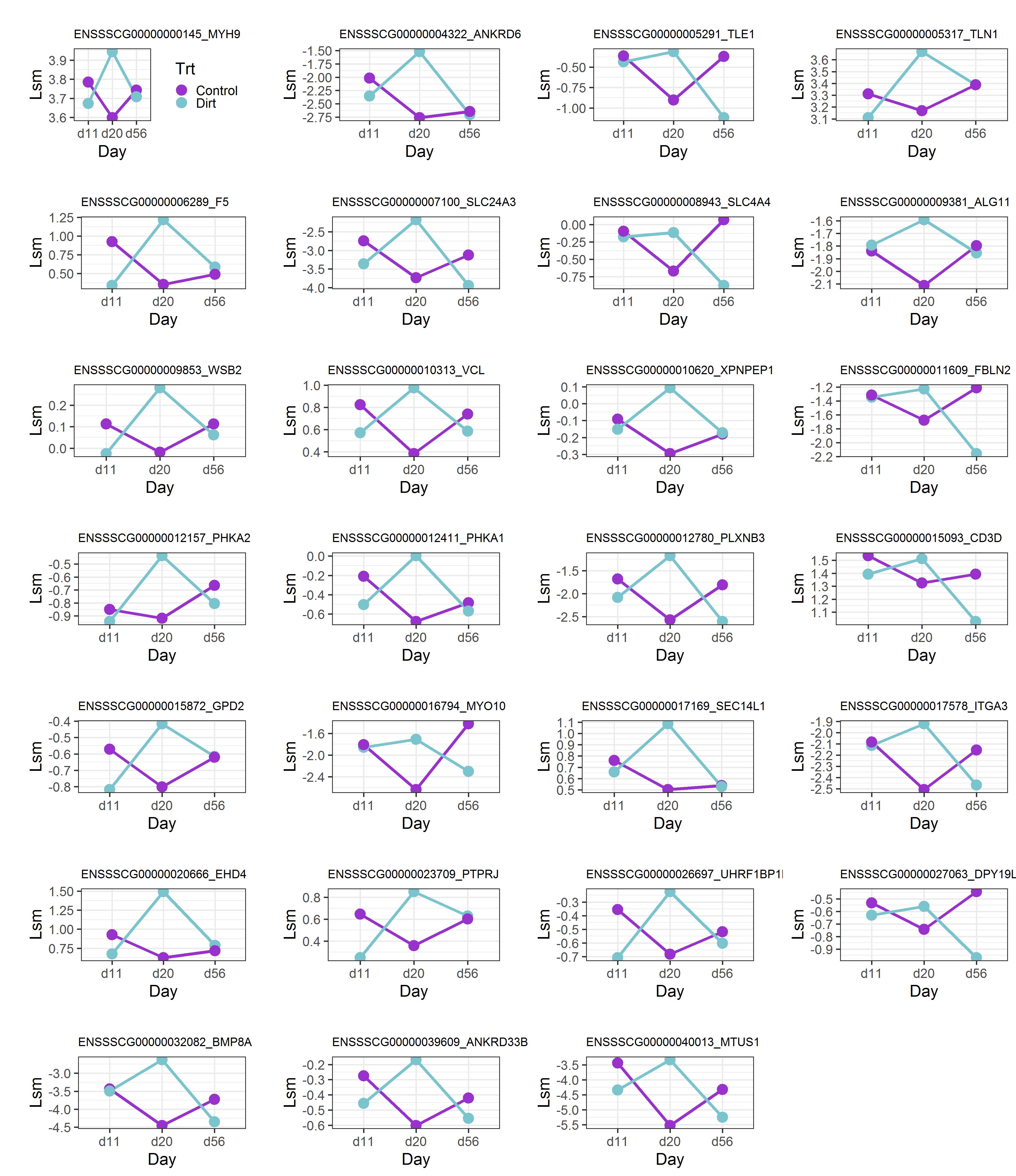


**Supplementary Figure S1.** Changes in relative gene expression (least square means) of 27 differentially expressed genes when comparing the topsoil to control treatment. These genes exhibit similar patterns of gene expression across time-points, with animals exposed to topsoil exhibiting an increase in expression from d 11 to d 20 and decrease at d 56. Control animals exhibit the opposite trend in gene expression patterns. Turquoise lines represent topsoil treatment group and purple lines represent the control group.


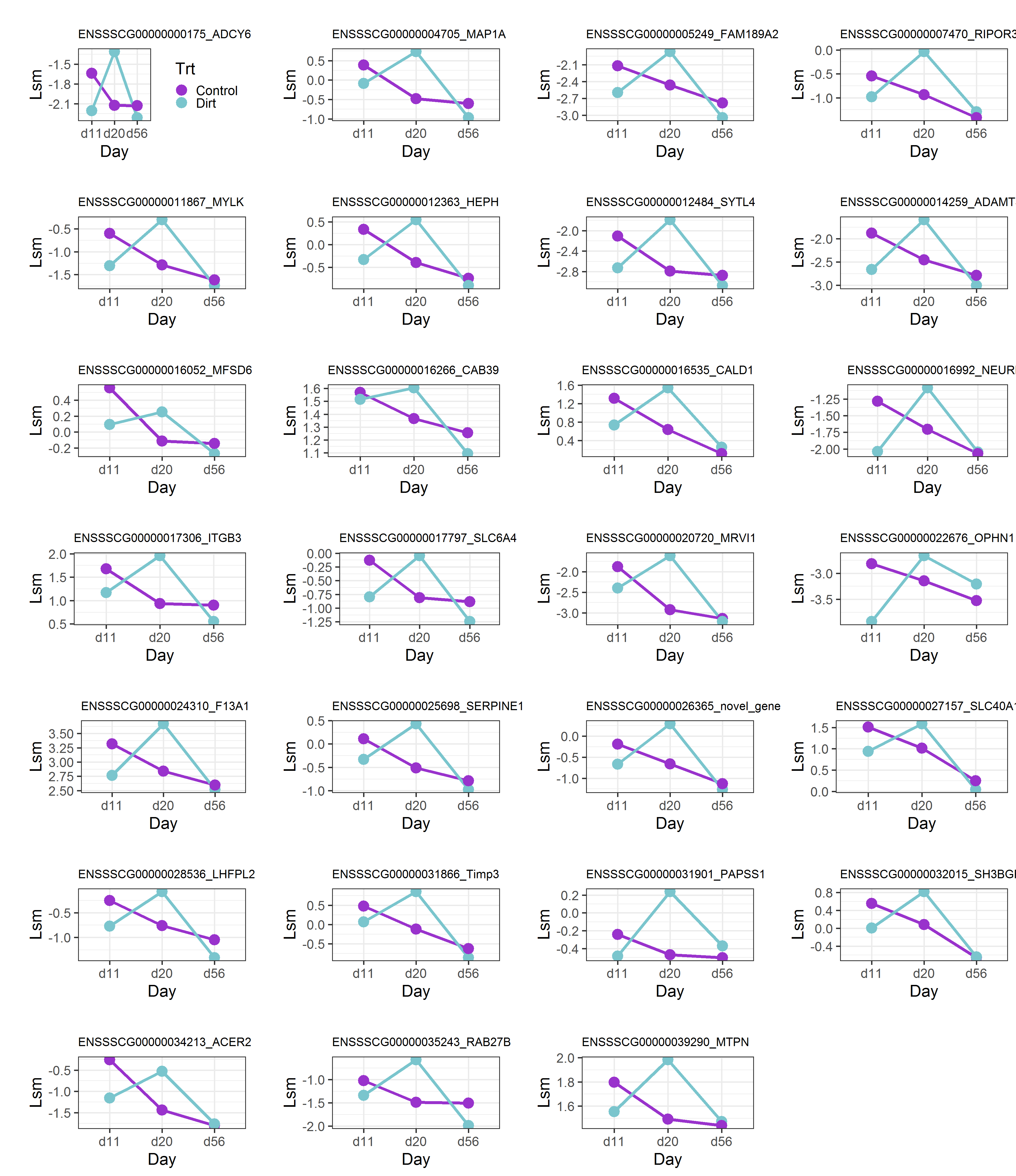


**Supplementary Figure S2.** Changes in relative gene expression (least square means) of 27 differentially expressed genes when comparing the topsoil to control treatment. These genes have similar patterns of expression across time-points, with animals exposed to topsoil exhibiting increased expression from d 11 to d 20 and decreased expression at d 56. Control animals exhibit decreased expression from d 11 to d 56. Turquoise lines represent topsoil treatment group and purple lines represent the control group.


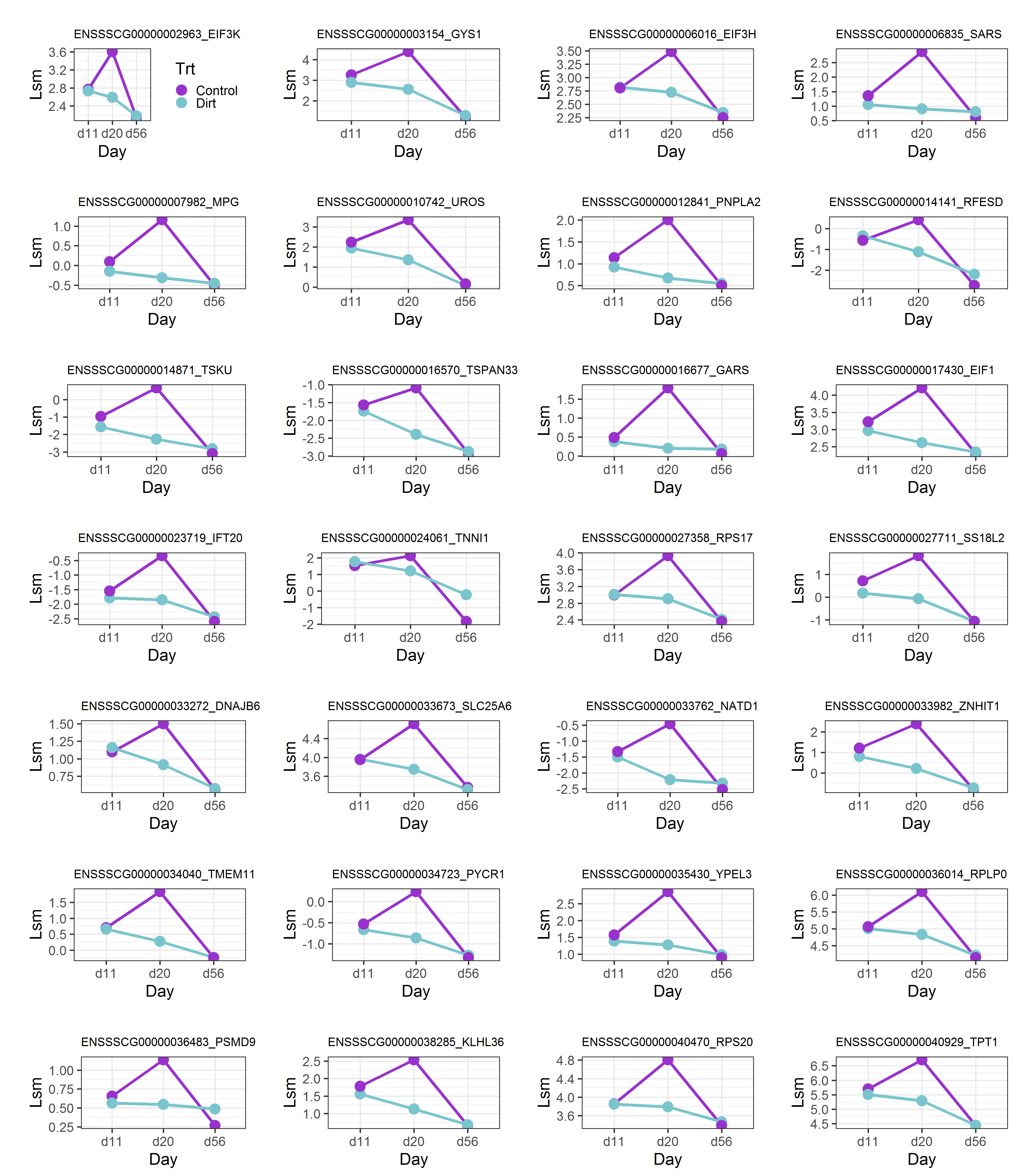


**Supplementary Figure S3.** Changes in relative gene expression (least square means) of 28 differentially expressed genes when comparing the soil to control treatment. These genes exhibit similar patterns of expression across time-points, with animals exposed to topsoil having increased expression from d 11 to d 20 and decreased expression at d 56. Control animals have decreased expression d 11 to d 56. Turquoise lines represent topsoil treatment group and purple lines represent the control group.


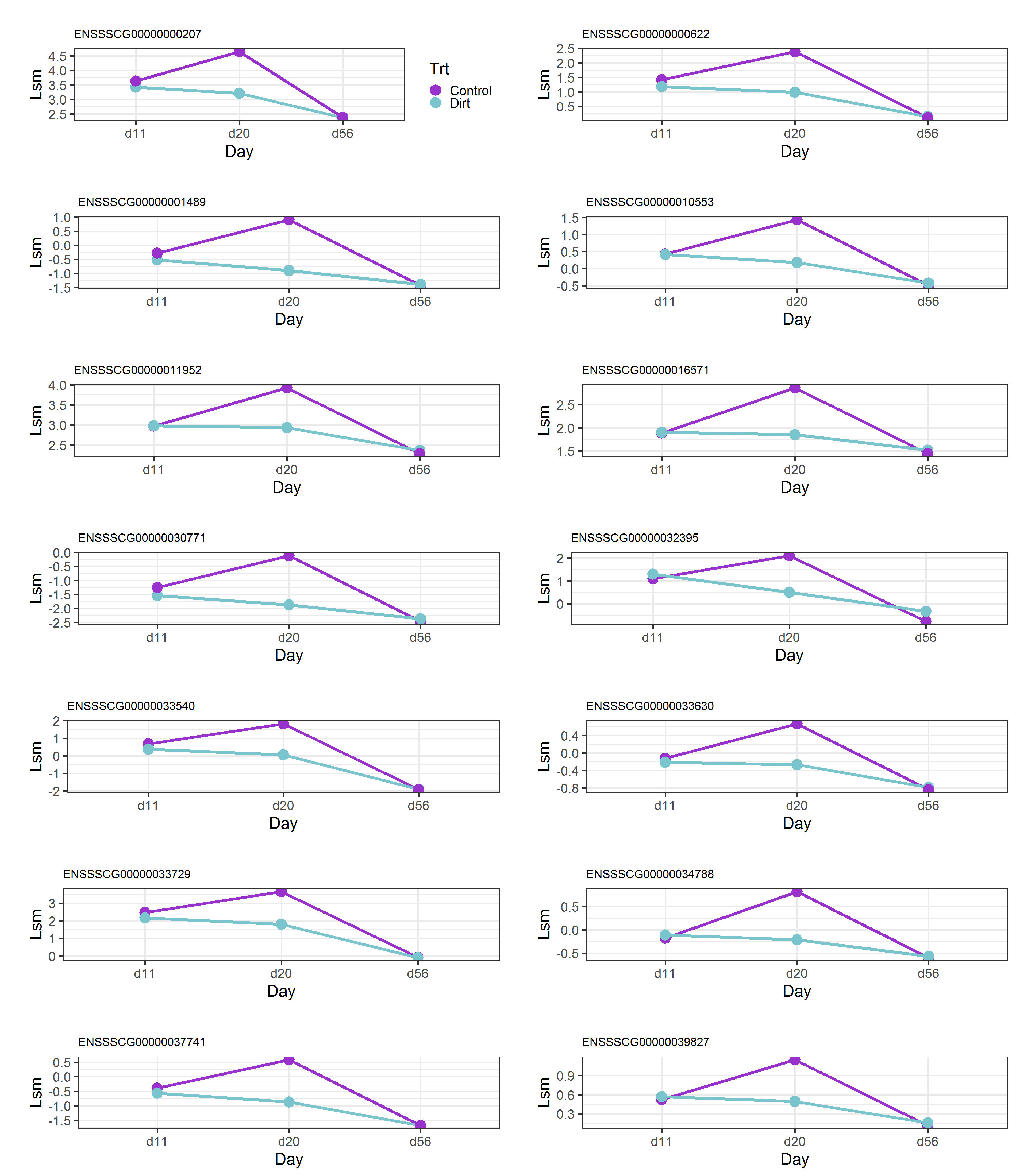


**Supplementary Figure S4.** Changes in relative gene expression (least square means) of 14 novel differentially expressed genes when comparing the topsoil to control treatment. The genes have similar patterns of expression across time-points. Animals exposed to topsoil have an increase of expression from d 11 to d 20 and decrease at d 56. Control animals have decreased expression from d 11 to d 56. Turquoise lines represent topsoil treatment group and purple lines represent the control group.


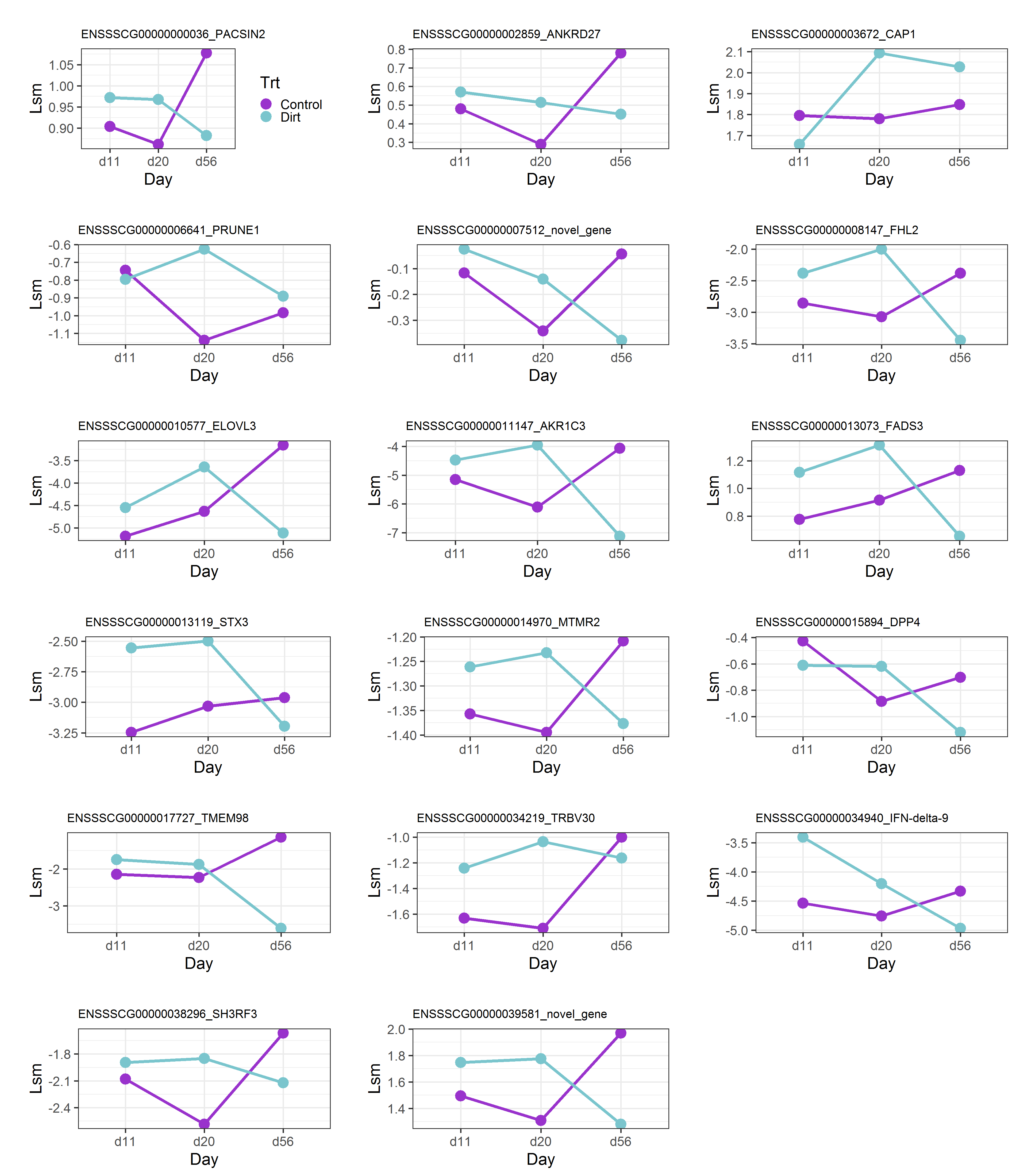


**Supplementary Figure S5.** Changes in relative gene expression (least square means) of 17 differentially expressed genes when comparing the topsoil to control treatment. Turquoise lines represent topsoil treatment group and purple lines represent the control group.


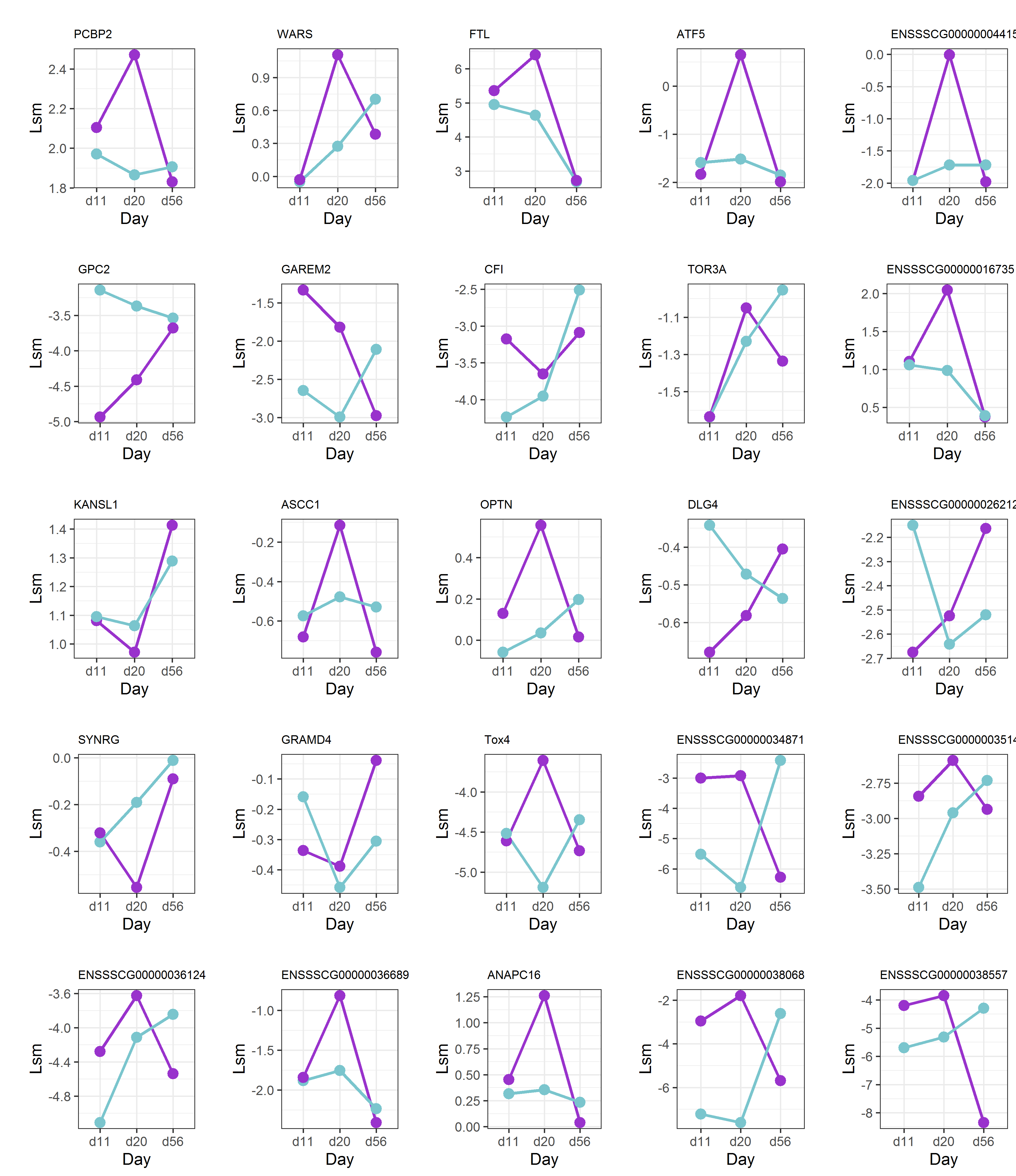


**Supplementary Figure S6.** Changes in relative gene expression (least square means) of 25 differentially expressed genes when comparing the topsoil to control treatment. Turquoise lines represent topsoil treatment group and purple lines represent the control group.

**
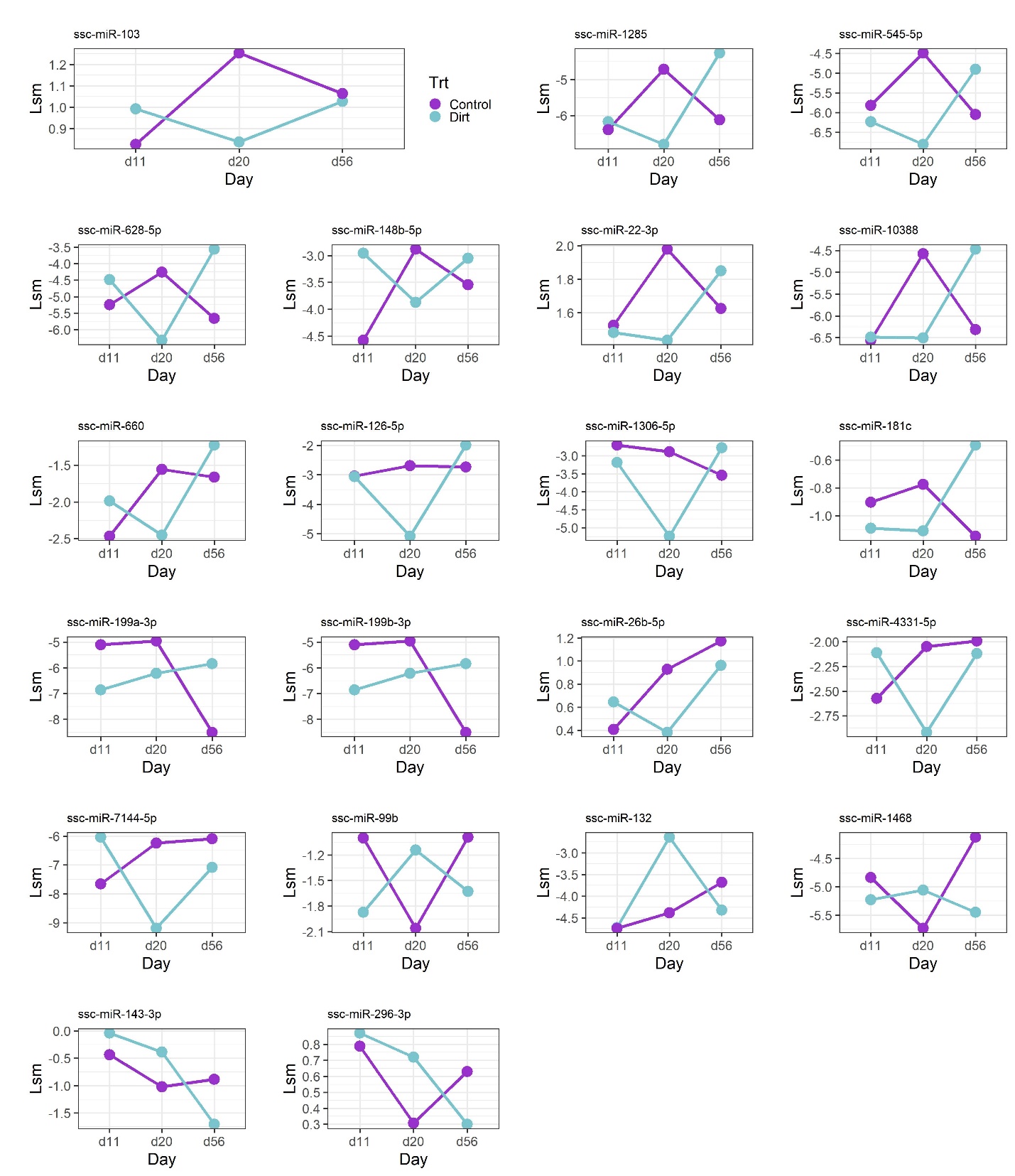
**

**Supplementary Figure S7.** Changes in relative gene expression (least square means) of 21 differentially expressed miRNAs when comparing the topsoil to control treatment. Turquoise lines represent topsoil treatment group and purple lines represent the control group.
